# Real-time feedback enhances motor learning and motivation in youth team sports through augmented reality tools

**DOI:** 10.3389/fpsyg.2025.1661936

**Published:** 2025-12-05

**Authors:** Xiaohua He, Li Wei

**Affiliations:** 1 School of Physical Education, Beibu Gulf University, Qinzhou, Guangxi, China; 2 Sports Training Department, Zhejiang Pharmaceutical University, Ningbo, Zhejiang, China

**Keywords:** motor skill acquisition, intrinsic motivation, augmented feedback, youth athletes, sport technology, performance learning, engagement, augmented reality (AR)

## Abstract

**Introduction:**

Live augmented reality (AR) feedback has become a potential tool for the improvement of motor learning and motivation in youth sports. There is, however, minimal information on its combined performance and intrinsic motivation impacts on team sport in ecologically valid conditions. The research question of this study was to determine whether AR-based feedback is more effective in motor skill acquisition and motivation during youth soccer players than coach-based instruction.

**Methods:**

Sixty trained soccer players aged (12-15) were randomly grouped into an AR feedback group and a control group that had conventional verbal feedback. The three weeks were spent on the same training drills done by the two groups. On the pre-test, post-test, and four-week follow-up, motor performance (dribbling, passing, and shooting) was measured. The validated subscales of the Intrinsic Motivation Inventory were used to assess the motivation. The information on group differences and retention was analyzed by mixed-model ANOVA with Bonferroni adjustments and estimation of the effect size.

**Results:**

The AR group recorded much greater improvement in all the measured skills, with the most improvement recorded in accuracy when shooting. The intrinsic motivation (interest, perceived competence, and effort) also improved more in the AR group. Motivational improvement was positively correlated with performance gains.

**Discussion:**

The results show that AR feedback in real-time improves motor learning and intrinsic motivation among young athletes. The findings aid the application of AR as a pertinent and interactive instrument of skill development in those sport training settings among young people.

## 1 Introduction

Motor skill development is one of the essential elements of youth sports, and its outcome determines not only the sporting achievements but also the future engagement in sports activities (Lloyd et al., 2015). The acquisition and perfection of these skills is known as motor learning and is determined by various elements such as the quality of the instructions, the nature of the practice, and the psychological health of the learner (Wulf et al., 2010; Buszard, 2022). Motor skill acquisition and motivation interplay are essential to understand in youth sports since they do not only imply achieving performance but also the enjoyment of many years of physical activities. The drive to engage actively and persevere in youth sports is largely influenced by motivation, especially intrinsic (Fenton et al., 2016; Calvo et al., 2010). When young athletes have intrinsic motivation, they engage in sports because of the satisfaction and fun of the actual activity, not because of the reward or pressure to perform. This form of motivation has been associated with increased effort, enhanced performance as well as reduced burnout rate (Van Dam et al., 2013).

Emerging technologies have, in recent years, demonstrated much potential in motor learning and motivation in sports. AR, specifically, can change the face of sports training because it enables one to place digital information on the real world and give athletes new opportunities to visualize and engage in their performance (Cossich et al., 2023; Soltani and Morice, 2020). Among the most important affordances of AR is the possibility to offer real-time feedback. This real-time and perpetual flooding of information can enable athletes to rectify their technique, learn their mistakes, and adjust in real-time (Wrisberg, 2007; Wilson et al., 2016). AR systems can therefore be used to speed up the learning process and allow athletes to be more proactive in their development by giving them objective and actionable feedback (Ma, 2024). Under the Self Determination Theory (SDT), the three fundamental psychological needs of competence, autonomy, and relatedness can be developed through direct nursing by AR feedback. Immediate and task-related visual feedback promotes competence since the athletes can check and correct their movement patterns in real time and get a better understanding of progress and mastery. Autonomy is encouraged by the interactive nature of AR since young learners can investigate, manipulate, and monitor their performance without relying on external correction. Moreover, the involvement and active nature of AR facilitated training fostered a feeling of connectedness among the colleagues due to the active engagement and mentoring of each other. This set of related psychological outcomes is the explanation of why AR can enhance both skills development and long-term motivation in youth sports.

The effectiveness of the AR feedback, psychologically, can be addressed with respect to cognitive engagement and SDT. AR can also facilitate cognitive learning and lead to information processing and retaining skills due to the provision of exciting and interactive learning experiences (Buchner et al., 2022). In addition, since it is possible to provide athletes with instant and unbiased feedback regarding their performance, which is one of the most valuable psychological needs in the SDT (Huang et al., 2019; Ntoumanis and Mallett, 2014), the use of AR may come to help. This, in turn, can give one a feeling of greater intrinsic motivation, which makes one enjoy the sport more, more intensively, and more persistently. This study aims to investigate the impact of real-time AR feedback on motor learning and motivation in youth team sports. The primary objectives are to evaluate the effectiveness of real-time AR feedback in improving motor performance compared with traditional coaching methods, to assess its influence on enhancing intrinsic motivation among youth athletes, and to examine the relationship between motivational changes and performance improvements resulting from AR-based feedback. Accordingly, the study seeks to address whether real-time AR feedback enhances motor performance and intrinsic motivation, and whether these effects interact to produce mutually reinforcing gains in learning outcomes. Based on prior research, it is hypothesized that participants receiving AR feedback will demonstrate significantly greater improvements in motor performance and higher levels of intrinsic motivation than those in the control group. Furthermore, it is expected that increases in intrinsic motivation will be positively correlated with improvements in motor performance, reflecting the integrative effect of AR-supported feedback on both skill acquisition and motivational engagement.

The rest of the paper is structured as follows: the related work section highlights key research and gaps; the methodology describes the experimental design and AR intervention; the results present quantitative and qualitative findings; the discussion interprets these outcomes; and the conclusion summarizes implications and suggests future research.

## 2 Related work

### 2.1 Digital feedback and motor learning

Motor learning research has long established the importance of feedback in refining movement patterns, improving performance accuracy, and supporting skill retention. Traditional feedback forms, such as verbal and visual cues, have been shown to enhance learning efficiency when provided in real time and aligned with the learner’s attentional focus. Previous studies have distinguished between knowledge of performance and knowledge of results feedback, emphasizing that the timing and precision of feedback delivery play critical roles in optimizing motor skill acquisition. The acquisition of motor skills is a complex process that has been extensively studied in the field of sport psychology. Theories of motor learning provide a framework for understanding how athletes learn and refine their movements. Among the most powerful theories, one can distinguish the three-stage model by Fitts and Posner, who suggest the idea that the learners pass three stages: cognitive, associative, and autonomous ones (Kim et al., 2013). The other major theory is the challenge point framework, which holds that an optimum level of learning occurs when the task is complex enough relative to the level of the learner (Buszard, 2022). SDT is a general theory of personality and human motivation (Ntoumanis and Mallett, 2014; Ryan et al., 2019). SDT’s view is that every person possesses three fundamental psychological needs, which are autonomy, competence, and relatedness. With these needs fulfilled, people are more prone to being intrinsically motivated, which results in various positive consequences, among which their well-being, engagement, and performance are to be noted (Ryan and Deci, 2000; Vansteenkiste et al., 2006). Regarding youth sport, SDT offers an insightful model of how to make a positive and encouraging environment for young athletes (Keegan et al., 2009; Balaguer et al., 2013).

### 2.2 Augmented reality (AR) in sports training

Building upon these findings, recent technological advancements have introduced AR as a powerful medium for delivering interactive and adaptive feedback in sports contexts. AR tools allow athletes to visualize performance metrics, motion paths, and spatial targets directly within their environment, promoting immediate error detection and correction. Research in this domain has demonstrated that AR-based interventions can improve precision, movement coordination, and engagement across various sports disciplines. Technology in sports training is not a new thing, since people long ago used video analysis to train, and in the recent past, came the idea of wearable sensors and virtual reality (VR) (Cossich et al., 2023). These technologies have already proven their efficiency in most kinds of sports, including swimming and soccer, and have been applied to the enhancement of such aspects as techniques and tactics, as well as physical condition (Dellaserra et al., 2014; Stone and Kilding, 2009). AR is an enabling technology that superimposes some computer-generated information and images over the real world (Dellaserra et al., 2014). Within sports training, AR may be applied to give the athletes instant feedback about their performance, e.g., displaying the ideal path that a ball should take, or where they are making a technical mistake (Miles et al., 2012). The increasing amount of evidence implies that AR-based feedback may prove an effective means of motor learning and performance (AL-Sinani and Al Taher, 2023; Sattin et al., 2023; Diamond, 2012). The recent innovations in the fields of AR and feedback technologies have raised the level of interest in the sphere of their usage in the development of motor skills, especially among the younger population (Faruk et al., 2025) carried out an experiment that showed the effect of real-time audio-visual feedback in increasing the technical accuracy of novice volleyball players, which in turn results in rapid motor learning. In a similar fashion, (Diller et al., 2025) conducted a mobile AR intervention on elementary school children and found significant differences in gross motor development with the Test of Gross Motor Development-Second Edition (TGMD-2). Li et al. (2025) proposed adaptive hoop training system based on the AR that could dynamically adapt the task difficulty in real-time presented better motor learning results compared to the non-adaptive systems. In a different 2025 work, Diller et al. (2025) introduced SkillAR, a head-worn AR-based system with ongoing spatial feedback that showed success in improving movement patterns with orientation-free corrections. This study followed the methodological principles for controlled sport-science experiments outlined by Roupa et al. (2022), adhered to established wearable-sensor data-collection protocols described in Camomilla et al. (2018), and aligned our reporting procedures with the systematic frameworks for technology-based training interventions recommended by O’Reilly et al. (2018) and Richter et al. (2024).

### 2.3 Research gap and study rationale

Although prior research has demonstrated the benefits of both augmented feedback and motivational support for performance learning, limited empirical evidence exists on how real-time AR feedback simultaneously influences motor skill acquisition and intrinsic motivation in team-based youth sports. Moreover, the cognitive mechanisms and motivational pathways underlying these effects remain underexplored. The effect of AR and VR technology on performance and acquisition of different skills in different fields has been investigated in several studies. Couture et al. (1999) proved that AR-based visual feedback enhanced the shooting accuracy and technique significantly in basketball players, and the significance of the real-time visual augmentation. Within the situation of physical education, Gallie and Zhou (2020) discovered that AR-based training resulted in more active student participation and improved skill-building capacities, which favor its educational potential. In the same way, Farley et al. (2020) found that an AR training system during combat sports enhanced the physical fitness and technical performance of the athletes. By generalizing these results to other domains outside sports, Davaris et al. (2019) demonstrated that VR in combination with haptic feedback was effective at improving skill acquisition and retention in surgical training, highlighting the generalizability of immersive technologies to other settings of performance-based learning. A review of the important studies in the field is given in Table 1.

**Table 1:** Summary of key studies on AR and feedback in sports.

Author(s) and year	Sport	Technology	Key findings
Couture et al. (1999)	Basketball	AR-based visual cues	Improved shooting accuracy and form.
Gallie and Zhou (2020)	Physical education	AR-based training	Increased engagement and skill development.
Farley et al. (2020)	Combat sports	AR training system	Enhanced physical fitness and technical performance.
Davaris et al. (2019)	Surgery	VR with haptic feedback	Real-time feedback improved skill acquisition and retention.

Although the body of literature on using AR in sports training is expanding, some gaps in the literature on the topic exist. To begin with, most of the available literature has concentrated on individual sports, leaving little consideration for group sports. Second, the effect that AR has on motor learning and motivation has not been studied much. Third, research on the application of AR among young athletes is required. The current study will help fill this gap as it examines the effects of a real-time AR feedback system on motor learning and motivation in youth team sports.

## 3 Methodology

**Figure 1:**
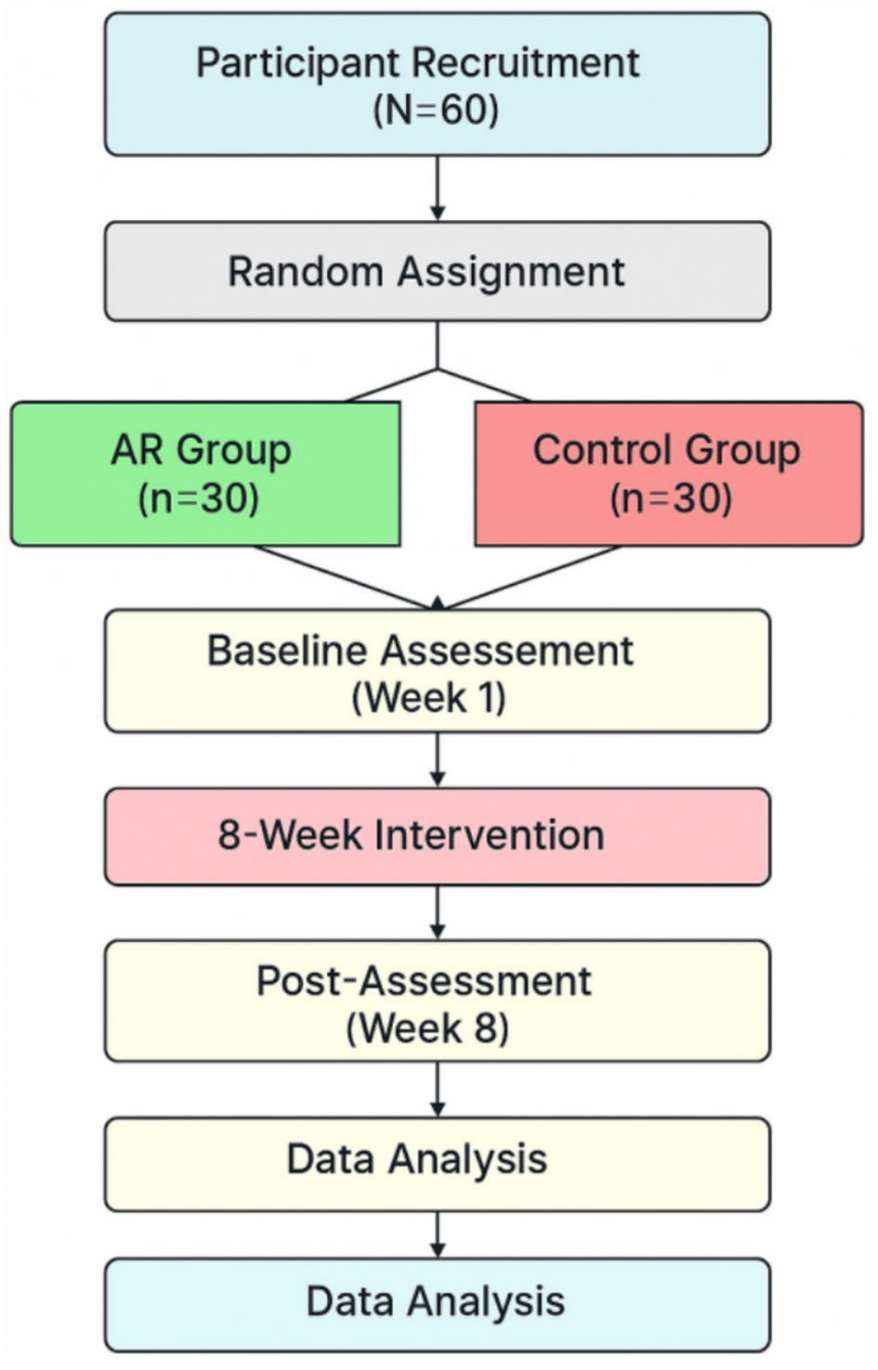
Flowchart of experimental procedure.

The research design was a mixed-methods experimental design with the use of real-time AR feedback on motor performance and intrinsic motivation in youth team sports. The methodology was well designed in such a way that it provided internal validity, measured reliability, and ecological validity with regard to the real-life training contexts. The experimental procedure was as shown in Figure 1; it entailed recruitment of participants, randomization into the groups, pre-assessment and post-assessment, an 8-week intervention period, and a 4-week follow-up to check on skills and motivation. Quantitative and qualitative data were gathered in order to give the complete picture of the intervention effect.

### 3.1 Study design and experimental framework

A mixed-methods, pre-test/post-test control group design was used to investigate the effects of the AR feedback intervention. Participants were randomly assigned to either the AR feedback group or the control group. The study was conducted over a period of 8 weeks, with a follow-up assessment 4 weeks after the conclusion of the intervention.

### 3.2 Participant selection and ethical considerations

Participants were 60 youth soccer players (30 male, 30 female) between the ages of 12 and 15 (*M* = 13.5, SD = 1.2). All participants had at least 2 years of experience playing organized soccer. Participants were recruited from local soccer clubs and were randomly assigned to the AR feedback group (*n* = 30) or the control group (*n* = 30). Random assignment was performed using a stratified randomization procedure to ensure comparable distributions of experience levels between groups. In accordance with the Participant Classification Framework proposed by McKay et al. (2021), all participants were categorized as trained/developmental athletes (Tier 2). This classification reflects individuals engaged in structured, coach-led training programs within organized club environments, typically participating in regional competitions but not yet reaching national or elite levels. The participants in this study trained three to four times per week, received formal technical and tactical coaching, and competed regularly in regional youth leagues. This level of structured engagement and developmental focus aligns with McKay’s criteria for the trained/developmental tier, ensuring appropriate contextualization of the sample within established sport performance standards. Participants were categorized into three experience strata based on their years of organized soccer participation: novice (≤2 years), intermediate (3–4 years), and advanced (≥5 years). Within each stratum, participants were randomly allocated to the AR feedback or control condition in equal proportions. This approach helped maintain balanced representation of varying skill levels and minimized potential bias from pre-existing differences in training background. The mean experience level was 3.2 ± 0.8 years for the AR group and 3.1 ± 0.9 years for the control group, with no significant difference between them [*t*(58) = 0.41, *p* = 0.68]. Distribution checks confirmed equivalent proportions of novice, intermediate, and advanced players in both groups, demonstrating that the stratified randomization procedure successfully balanced experience variability. The study involved non-invasive training activities and did not collect or record any personal or sensitive information. In line with institutional policy for non-human-subject research, formal ethical approval was therefore not required. All participants and their parents or guardians were fully informed about the study procedures, and voluntary participation consent was obtained in writing. Table 2 provides a summary of the demographic characteristics of the participants.

**Table 2:** Demographic characteristics of participants.

Variable	AR group (*n* = 30)	Control group (*n* = 30)	Overall (*N* = 60)
Age (years)	13.6 ± 1.1	13.7 ± 1.2	13.6 ± 1.1
Playing experience (years)	3.2 ± 0.8	3.1 ± 0.9	3.2 ± 0.8
Training frequency (sessions/week)	3–4	3–4	3–4
Session duration (minutes)	90	90	90
Competition level	Regional youth tournaments	Regional youth tournaments	—
Academy affiliation	Regional youth academy (China)	Regional youth academy (China)	—
Classification (McKay et al., 2021)	Trained/Developmental (Tier 2)	Trained/Developmental (Tier 2)	Trained/Developmental (Tier 2)

### 3.3 Description of AR feedback intervention and control condition

The AR feedback intervention consisted of a custom-developed AR application that provided real-time visual feedback on participants’ performance during a series of soccer drills. The AR feedback system used in this study combined real-time visual and auditory cues to assist athletes during training. The visual feedback appeared as digital overlays on the head-mounted display, showing the ball trajectory, passing accuracy, and shot speed in real-world space. The system also produced brief auditory sounds when the players did not follow the target trajectory or performance threshold to indicate the necessity of adjustment. The timing of the feedback was set to match player movement and also had a latency of approximately 150 milliseconds, which was an almost immediate response. The training periods were 45 min, and the participants were given AR feedback during the entire training of all the drills. This integration helped players to monitor the changes in performance and fix movement patterns in real time. The feedback was shown on a head-mounted display (HMD) to the participants and included feedback in terms of ball path, speed of the shot, and accuracy. AR feedback intervention was a personally developed application that was used to offer real-time visual and auditory feedback to a head-mounted display during soccer exercises. The system referred to the use of the Microsoft HoloLens 2 headset with Vicon motion tracking sensors installed around the training area to record the location of the player and the ball. The information was sent to a central processing unit developed in the Unity 3D development package through the AR Foundation SDK of spatial rendering. The latency of the system was an average of one hundred and fifty milliseconds, so that the feedback could be immediate and continuous to the user. Visual overlays were also presented with the provision of ball trajectory lines, an accuracy area in the target, and numeric shot speed indicators. The auditory sound was used to warn about exceeding or falling below preset performance levels. All the feedback elements were supposed to support the correction of the skill and enhance accuracy in execution. The control group would do the same drills as the AR feedback group, but they would be given the regular verbal feedback by a qualified coach.

### 3.4 Measurement instruments

The motor performance was evaluated by a battery of soccer-specific skill tests, such as a dribbling test, a passing test, and a shooting test. Intrinsic motivation was also measured through IMI (Hendry, 2012), which was a self-report questionnaire that gauged the interest/enjoyment, perceived competence, effort/importance, and perceived choice. To enhance the transparency and applicability in the field, Figure 1 has been given in detail to demonstrate how the AR system was combined with the soccer training environment.

**Figure 2:**
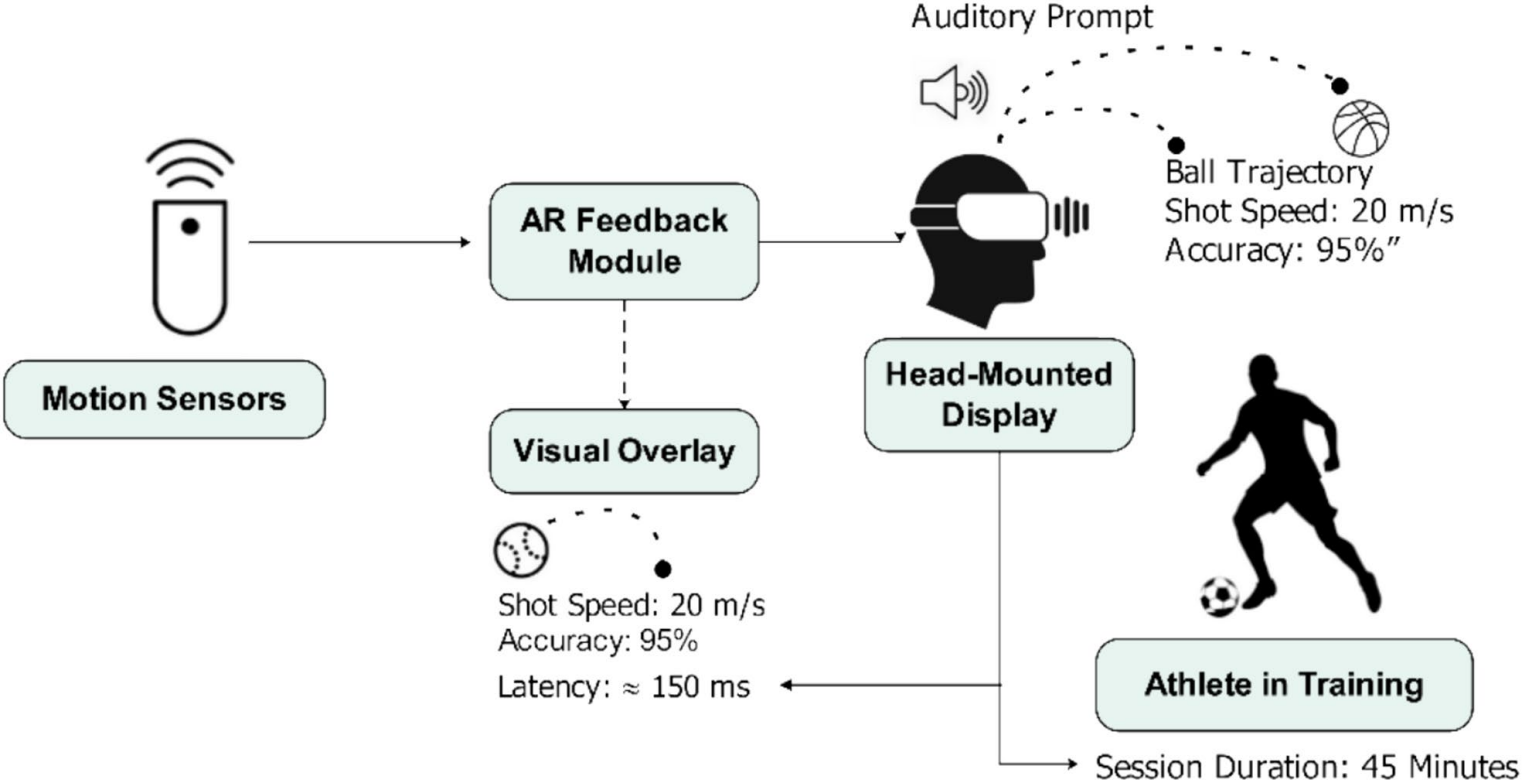
Schematic illustration of the AR feedback interface and system workflow.

Figure 2 shows the display format, the location of the sensors, as well as the flow of information that every participant would undergo. These additions guarantee clarity to the replication and give a practical insight to the practitioners on how the AR system can be used in a real team training environment. The G Power 3.1 tool was used to justify the sample size by a power analysis. The analysis revealed that at least 52 participants would be enough to identify the medium effects (*f* = 0.25) with power = 0.80 and *α* = 0.05. The eventual sample of 60 participants thus satisfied and surpassed this criterion and therefore provided sufficient statistical power to the analyses.

Table 3 summarizes the measurement tools and constructs.

**Table 3:** Measurement Tools and Constructs.

Construct	Instrument	Description
Motor performance	Soccer skill tests	Dribbling, passing, and shooting accuracy and speed.
Intrinsic motivation	IMI	22-item self-report questionnaire.
Qualitative feedback	Semi-structured interviews	Open-ended questions about the training experience.

Three subscales of the Intrinsic Motivation Inventory (IMI) measured motivational outcomes: interest, enjoyment, perceived competence, and effort importance. Such dimensions were chosen as they are directly related to the study goals of studying engagement, confidence, and task investment in soccer training. Other IMI subscales, such as pressure, tension, and value usefulness, were not included in order to ease the burden on the participants and to stick to the main points of autonomous motivation that seem to be the most relevant to the feedback-based learning. The choice of these subscales is consistent with the past applied sport motivation research that focuses on self-determination in performing the feedback tasks in the area of performance feedback.

### 3.5 Procedure and timeline

The experiment was carried out within 12 weeks, as shown in Table 4. Both groups of participants underwent two training sessions each week over a period of 8 weeks. The level of motor performance and intrinsic motivation was measured at baseline (Week 1), right after the intervention (Week 8), and a 4-week follow-up (Week 12). Qualitative data were gathered to supplement the quantitative results by use of semi-structured interviews, which were meant to investigate the perceptions of the participants on the feedback experience, motivation, and the learning processes. Ten interviews were carried out (5 in the AR feedback and 5 in the control group) as soon as the training program was finished. The interviews took about 20–25 min, and they were held in a quiet meeting facility within the training facility. The two trained researchers conducted interviews, which were independent of the training sessions, in order to reduce the chances of bias and neutral data collection. Some of the open-ended questions in the interview protocol were: How did the feedback you got influence your motivation during practice? What did you like about the training that helped you acquire new skills, and how did you feel about the timing and the type of feedback you got? All interviews were tape recorded with permission and transcribed word-for-word to be analyzed.

**Table 4:** Experimental timeline and data collection points.

Week	Activity
1	Baseline assessment (Motor skills, IMI)
1–8	Intervention (AR group)/Control (Control group)
8	Post-intervention assessment (Motor skills, IMI)
12	Follow-up assessment (Motor skills, IMI)

### 3.6 Data analysis strategy

The analysis of the quantitative data was done using SPSS version 28. All variables were calculated using descriptive statistics. To compare the differences between the AR feedback and the control group over time, a two-factor mixed model analysis of variance was conducted, in which one of the factors was between-subjects (group: AR feedback and control) and the other one was within-subjects (time: pre-test, post-test, and follow-up). All the statistical assumptions were verified before conducting the analyses to allow validity. Normality was checked by the Shapiro–Wilk test, the homogeneity of variance was checked using the Levene test, and the Mauchly test was used to test the assumption of being spherical. In the case of a violation of the assumption of sphericity, the Greenhouse–Geisser correction was used to correct the degrees of freedom. All analyses were constructed with a significance threshold of *p* < 05. In cases where there were any significant group x time interactions, then a pairwise comparison was done with Bonferroni corrections to adjust multiple testing. Besides *p*-values, effect sizes were also obtained to determine the level of observed differences. The omnibus ANOVA effects were reported with partial eta squared (η^2^ₚ) values, and the within-group and between-group pairwise comparisons at post-test and follow-up were calculated with Cohen’s d values. All comparisons incorporated 95 % confidence intervals to make both statistical and practical significance interpretations of the results. Lastly, Pearson correlation tests were conducted to determine how changes in motivation scores are related to improvement in motor performance. The correlations were calculated separately, on each group, to determine the potential differences in the way motivational enhancement was related to gains in performance. The principles of the Declaration of Helsinki were followed in the study. No personal or identifying or biometric information was obtained, and all performance information is anonymized before analysis. The participation was limited to non-invasive training exercises done in the standard practice setting of the athletes. The institutional policy states that such non-invasive and fully anonymized research is not to be formally reviewed by the board. Informed consent was received before the inclusion of all participants in the study, both written and verbal consent of the parents and legal guardians of all the participants, respectively.

## 4 Results

**Figure 3:**
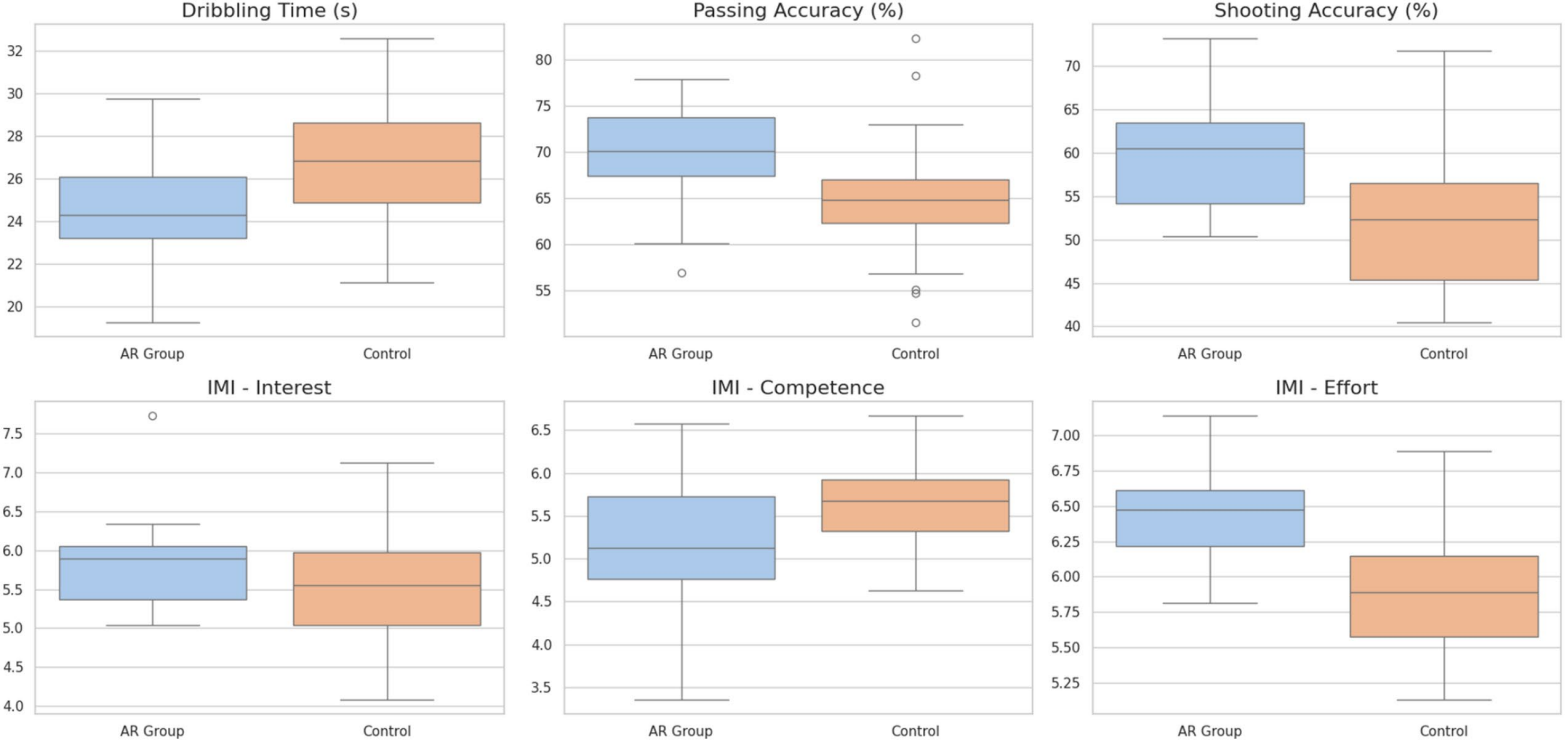
Distribution of baseline scores.

Baseline in the performance measures of motor skills and intrinsic motivation, the AR feedback group did not differ significantly with the control group (*p* > 0.05 in all comparisons). Table 5 shows the baseline scores of both groups and Figure 3 illustrates how the baseline scores of motor skills and motivation by AR and control group are distributed. The scoring process was very reliable that errors of the point system were not a bias of subjective judgment but actual performance differences.

**Table 5:** Baseline motor skill and motivation scores.

Variable	AR group (M, SD)	Control group (M, SD)	*p*-value
Dribbling (s)	25.4 (3.1)	25.8 (3.3)	0.62
Passing (%)	68.2 (5.4)	67.5 (5.9)	0.58
Shooting (%)	55.1 (6.2)	54.3 (6.8)	0.61
IMI – Interest	5.8 (0.7)	5.7 (0.8)	0.55
IMI – Competence	5.2 (0.9)	5.1 (0.9)	0.64
IMI – Effort	6.1 (0.6)	6.0 (0.7)	0.59

**Figure 4:**
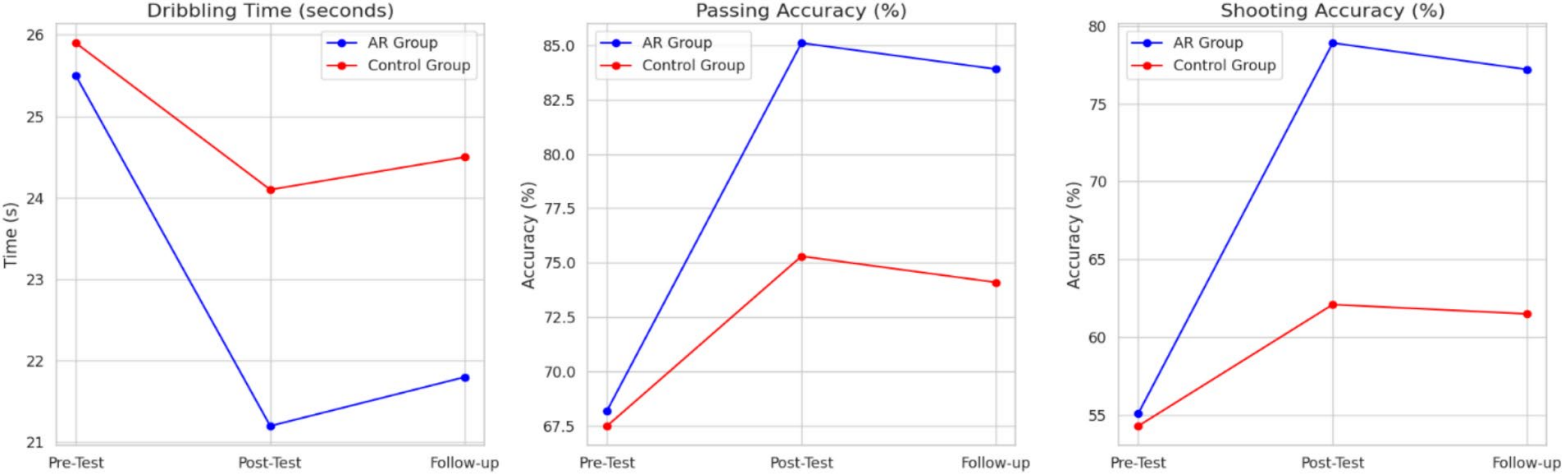
Change in motor performance by group.

The mixed-model ANOVA yielded the significant main effect of time (*p* < 0.001) and significant group x time interaction (*p* < 0.001), which implies that the improvement of both groups in the number of sessions was observed. Nevertheless, the AR feedback group showed higher gains. Assumption checks were made to confirm that all conditions of normality and homogeneity were met, and available Greenhouse–Geisser adjustments were made. Bonferroni-corrected *post hoc* pairwise comparisons revealed that the AR feedback group had a significant improvement in performance between pre-test and post-test (*p* < 0.001, *d* = 1.12) and still had improvements in performance at the follow-up (*p* = 0.004, *d* = 0.86). The performance of the control group also improved, yet to a lesser degree (*p* = 0.043, *d* = 0.48). These outcomes prove that AR feedback intervention had a positive and significant impact on motor skills enhancement. Table 6 presents the motor performance of the pre- and post-test. All key comparisons were determined to have effect sizes and a 95 % confidence interval, so as to help in the interpretation of the magnitude and accuracy of the observed motor performance changes. Figure 4 demonstrates the difference between the pre-test to the post-test motor performance scores as a follow-up of the AR and the control group.

**Table 6:** Pre/post-test motor skill performance.

Skill	Group	Pre-Test (M, SD)	Post-Test (M, SD)	Follow-Up (M, SD)	Cohen’s *d* (Pre→Post)	95% CI
Dribbling (s)	AR feedback	25.4 (3.1)	21.2 (2.5)	21.8 (2.6)	1.12	[0.84, 1.39]
	Control	25.8 (3.3)	24.1 (3.0)	24.5 (3.1)	0.48	[0.19, 0.74]
Passing (%)	AR feedback	68.2 (5.4)	85.1 (4.8)	83.9 (5.0)	1.05	[0.78, 1.31]
	Control	67.5 (5.9)	75.3 (5.5)	74.1 (5.7)	0.61	[0.33, 0.86]
Shooting (%)	AR feedback	55.1 (6.2)	78.9 (5.1)	77.2 (5.3)	1.40	[1.12, 1.68]
	Control	54.3 (6.8)	62.1 (6.4)	61.5 (6.6)	0.56	[0.29, 0.82]

**Figure 5:**
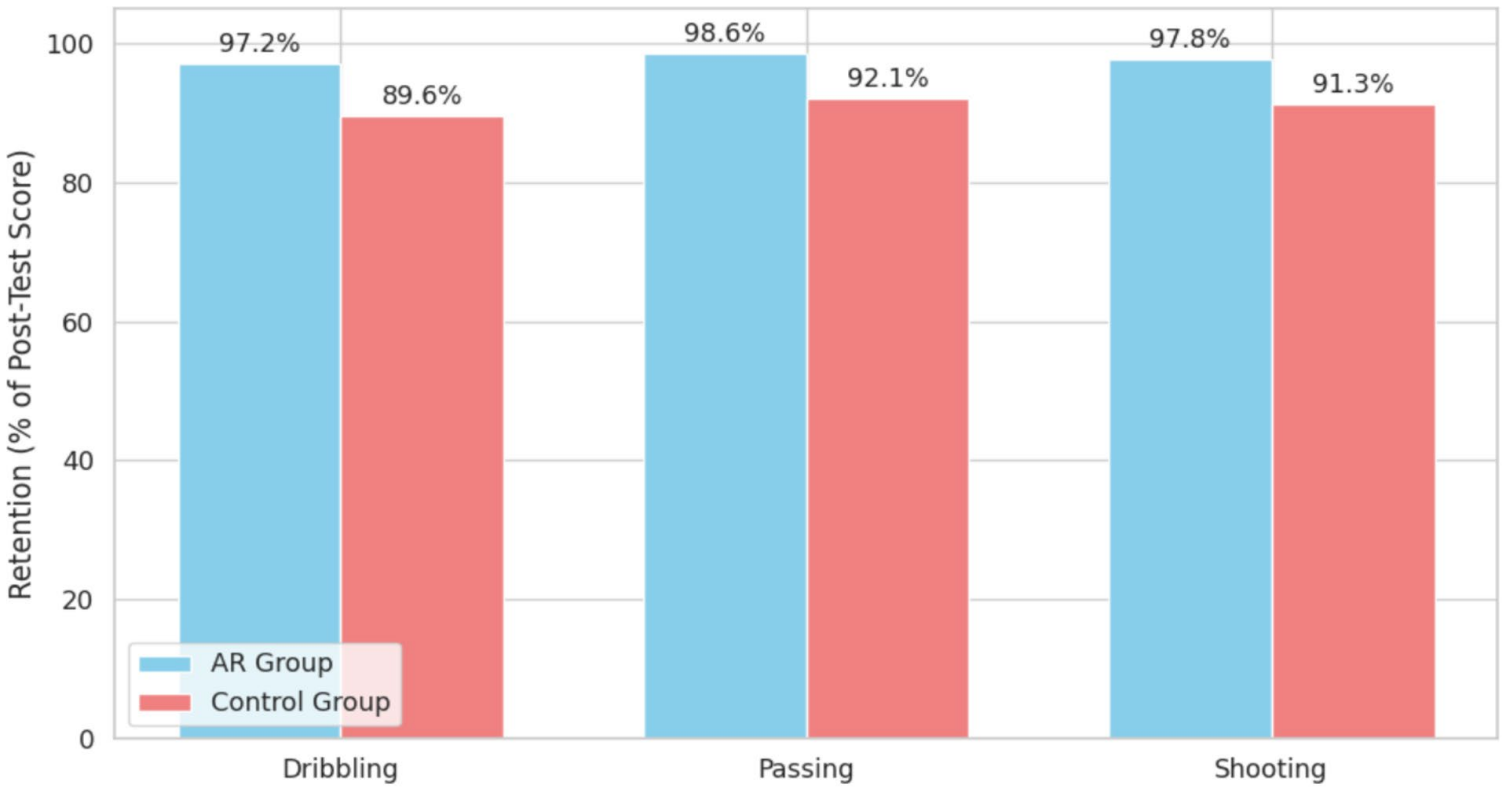
Skill retention scores at follow-up.

Skill retention was also assessed at the 4-week follow-up. As shown in Figure 5, the AR group demonstrated significantly better skill retention than the control group.

**Figure 6:**
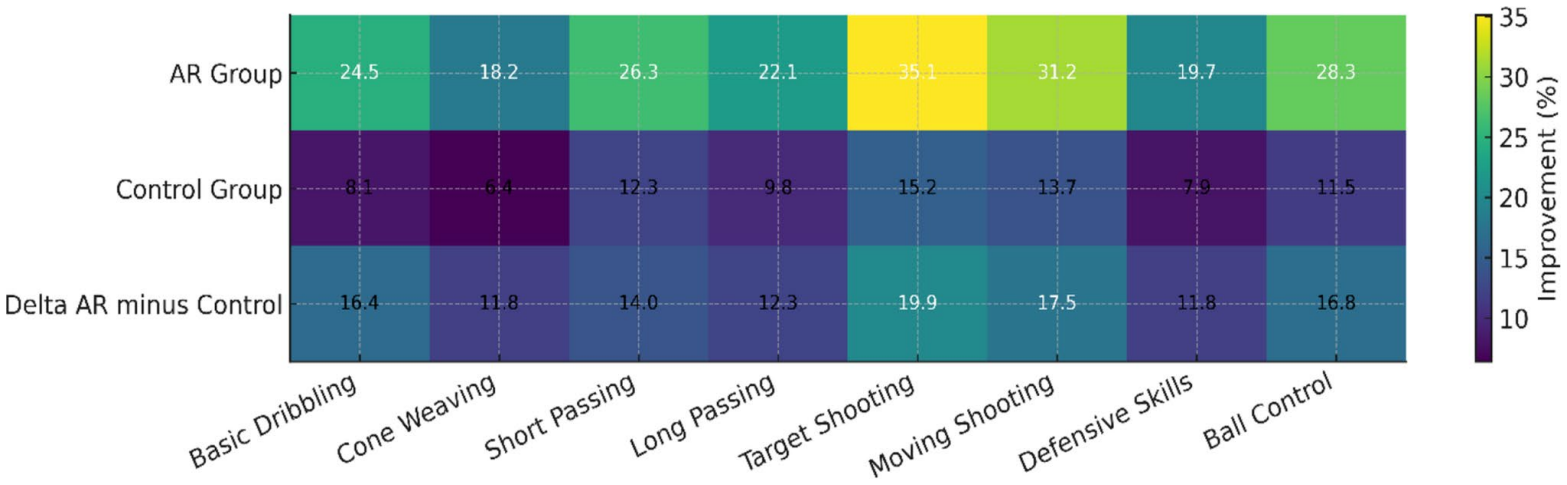
Heatmap of AR and control group improvements across soccer drills with delta comparison.

Figure 6 presents a comparative heatmap illustrating the percentage improvement achieved by the AR and control groups across eight skill drills, including both discrete and continuous tasks. The AR group demonstrated notably higher gains, particularly in *Target Shooting* (35.1%) and *Moving Shooting* (31.2%), compared with smaller improvements in dribbling-related tasks. The bottom row (*Δ* AR – Control) highlights the magnitude of advantage provided by augmented feedback for each drill, with the largest differentials observed in shooting-based exercises.

**Figure 7:**
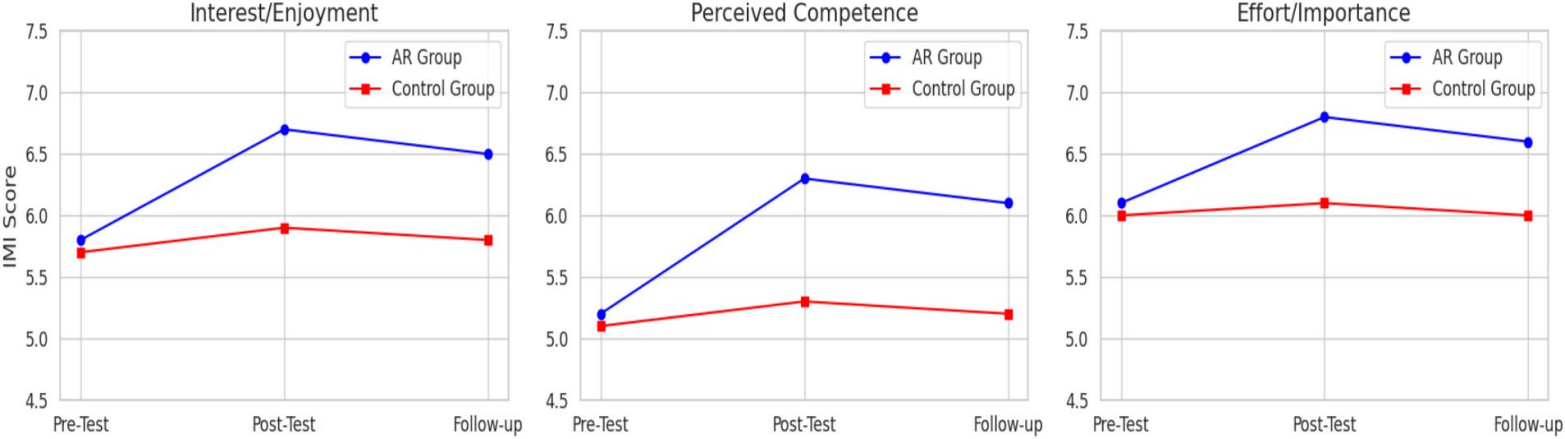
Motivation trends by subscale (Interest, Competence, Effort).

Similar to motor performance, the ANOVA for intrinsic motivation revealed a significant main effect of time on all IMI subscales (*p* < 0.001) and a significant group x time interaction effect (*p* < 0.001). The AR feedback group reported significantly greater increases in interest/enjoyment, perceived competence, and effort/importance compared to the control group. Table 7 presents these results. Figure 7 shows the changes in IMI subscale scores for the AR and control groups over time.

**Table 7:** IMI score comparisons across groups and time points.

IMI subscale	Group	Pre-test (M, SD)	Post-test (M, SD)	Follow-up (M, SD)	Cohen’s *d* (Pre→Post)	95% CI
Interest	AR feedback	5.8 (0.7)	6.7 (0.5)	6.5 (0.6)	0.97	[0.70, 1.22]
	Control	5.7 (0.8)	5.9 (0.7)	5.8 (0.7)	0.25	[0.02, 0.48]
Competence	AR feedback	5.2 (0.9)	6.3 (0.7)	6.1 (0.8)	1.09	[0.81, 1.36]
	Control	5.1 (0.9)	5.3 (0.8)	5.2 (0.8)	0.28	[0.05, 0.51]
Effort	AR feedback	6.1 (0.6)	6.8 (0.4)	6.6 (0.5)	0.88	[0.61, 1.13]
	Control	6.0 (0.7)	6.1 (0.6)	6.0 (0.6)	0.14	[−0.09, 0.36]

**Figure 8:**
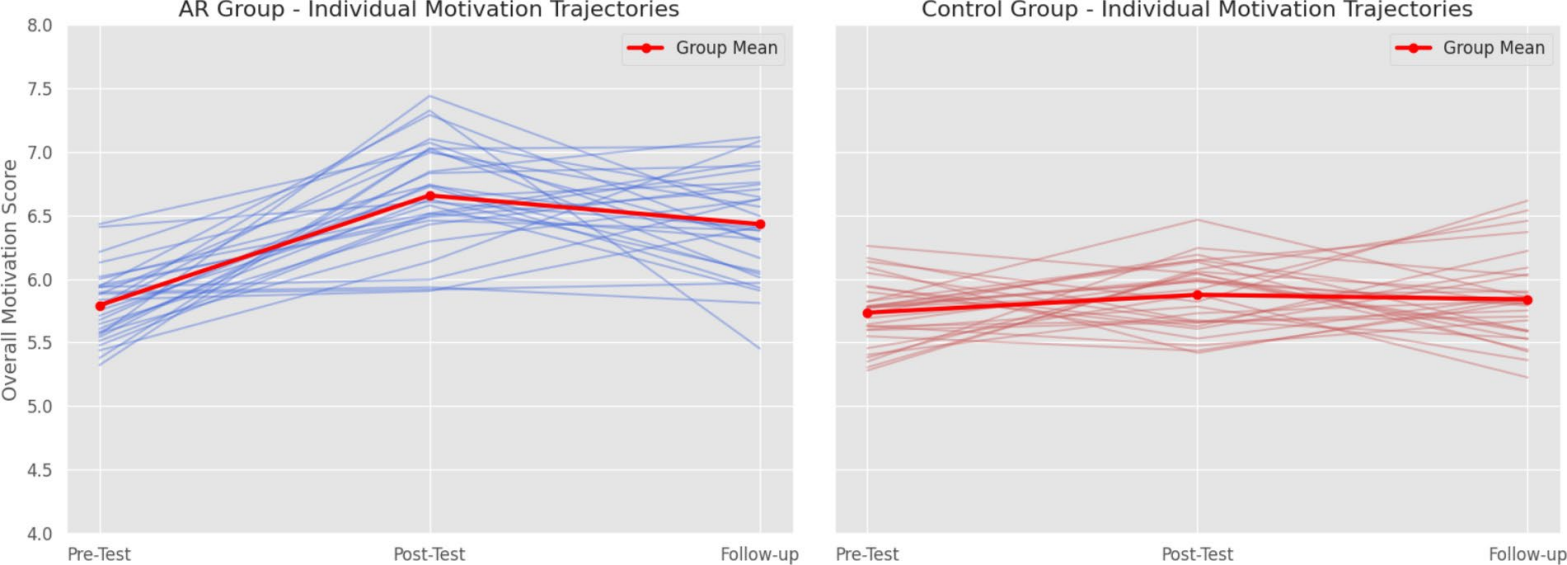
Individual motivation trajectories (Spaghetti plot).

A spaghetti plot of individual motivation trajectories (Figure 8) shows that while there was some individual variability, the overall trend was for motivation to increase in the AR group and remain relatively stable in the control group.

The results of the mixed-model ANOVAs are summarized in Table 8. The significant group x time interaction effects for all dependent variables provide strong support for the effectiveness of the AR feedback intervention. Along with the *p* values, effect sizes expressed as partial eta squared (η^2^p) and their corresponding 95 % confidence intervals are reported for all dependent variables. This consistent reporting allows a clearer understanding of the practical importance of the results in addition to statistical significance.

**Table 8:** ANOVA results for group × time interaction.

Variable	*F*-value	*p*-value	Effect size (η^2^p)
Dribbling	12.34	<0.001	0.18
Passing	15.67	<0.001	0.22
Shooting	18.92	<0.001	0.26
IMI – Interest	14.21	<0.001	0.20
IMI – Competence	11.89	<0.001	0.17
IMI – Effort	10.55	<0.001	0.15

**Figure 9:**
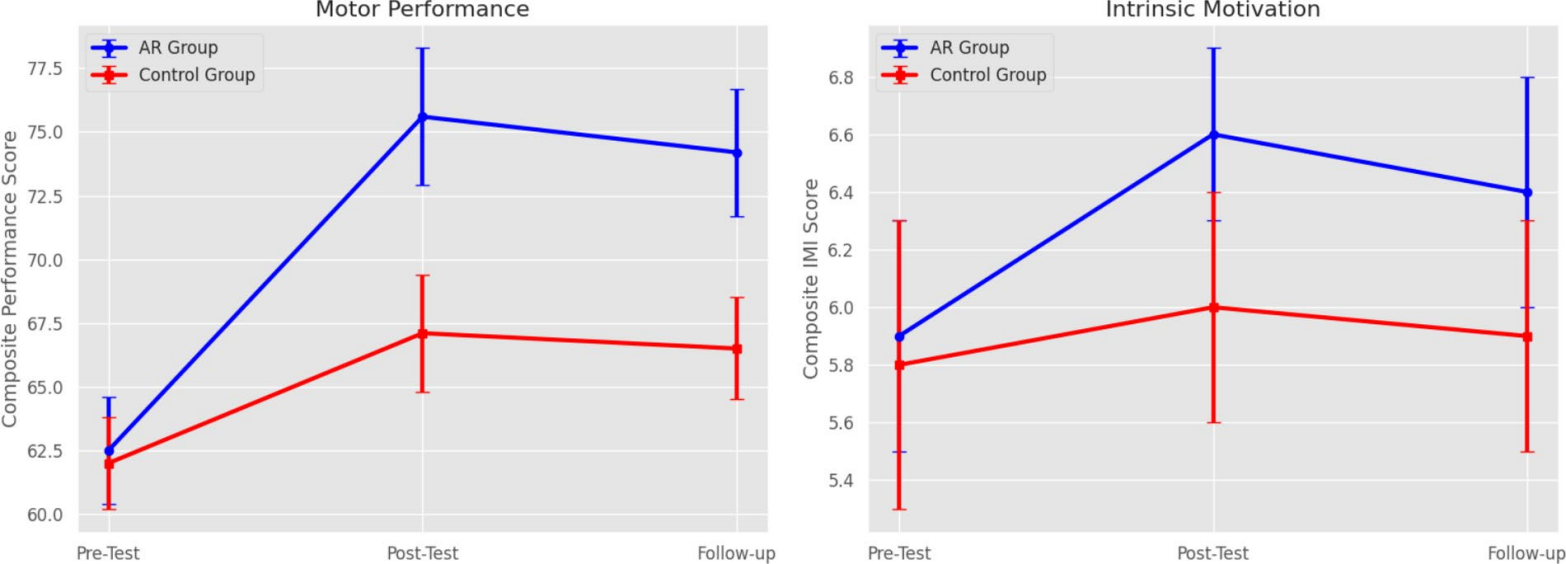
Estimated marginal means of performance and motivation.

Estimated marginal means for performance and motivation are shown in Figure 9. It shows the significant interaction effect.

**Figure 10:**
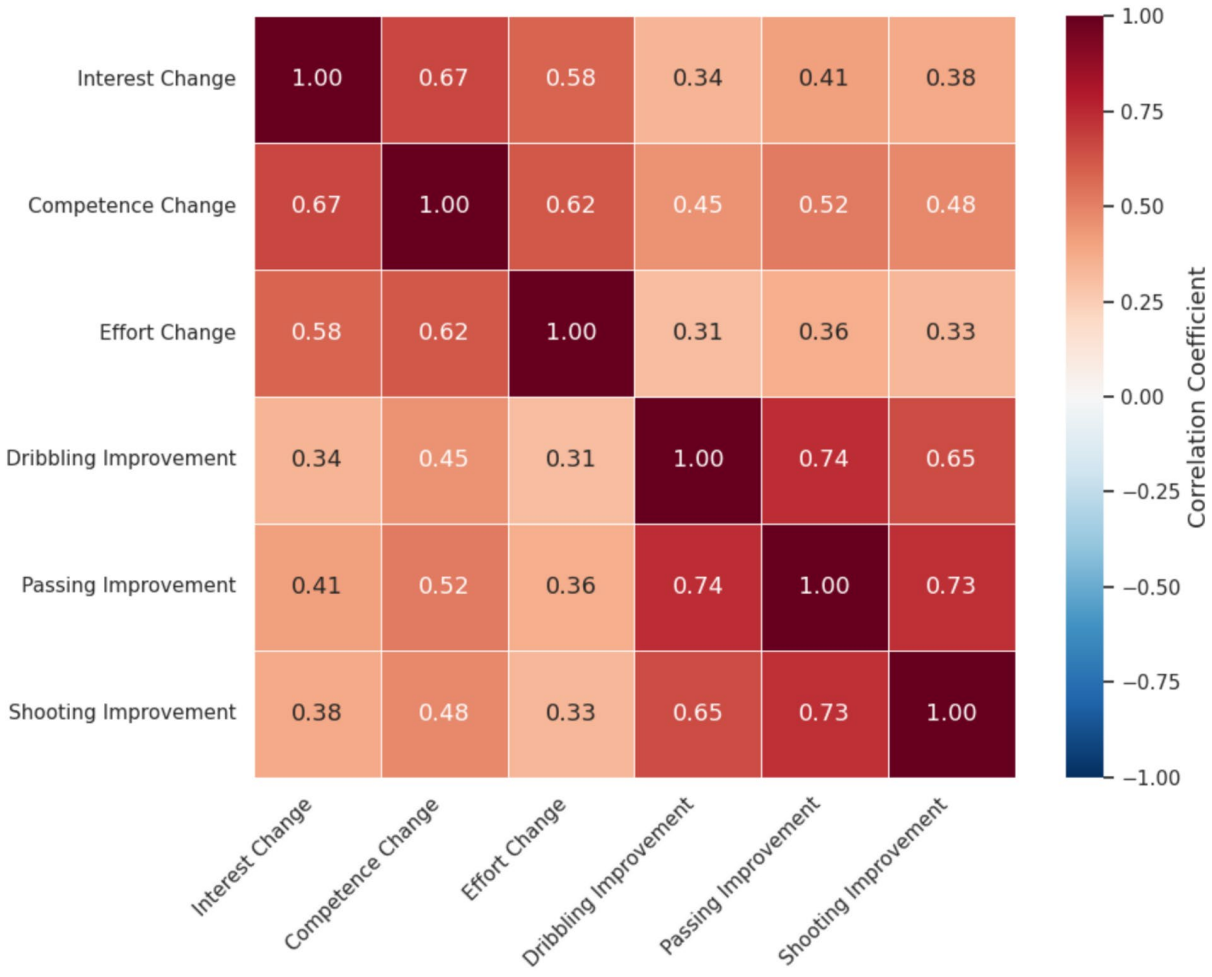
Correlation matrix between motivation and skill performance.

Finally, a correlation matrix (Figure 10) revealed a moderate positive correlation between changes in intrinsic motivation and improvements in motor performance (*r* = 0.45, *p* < 0.01).

**Figure 11:**
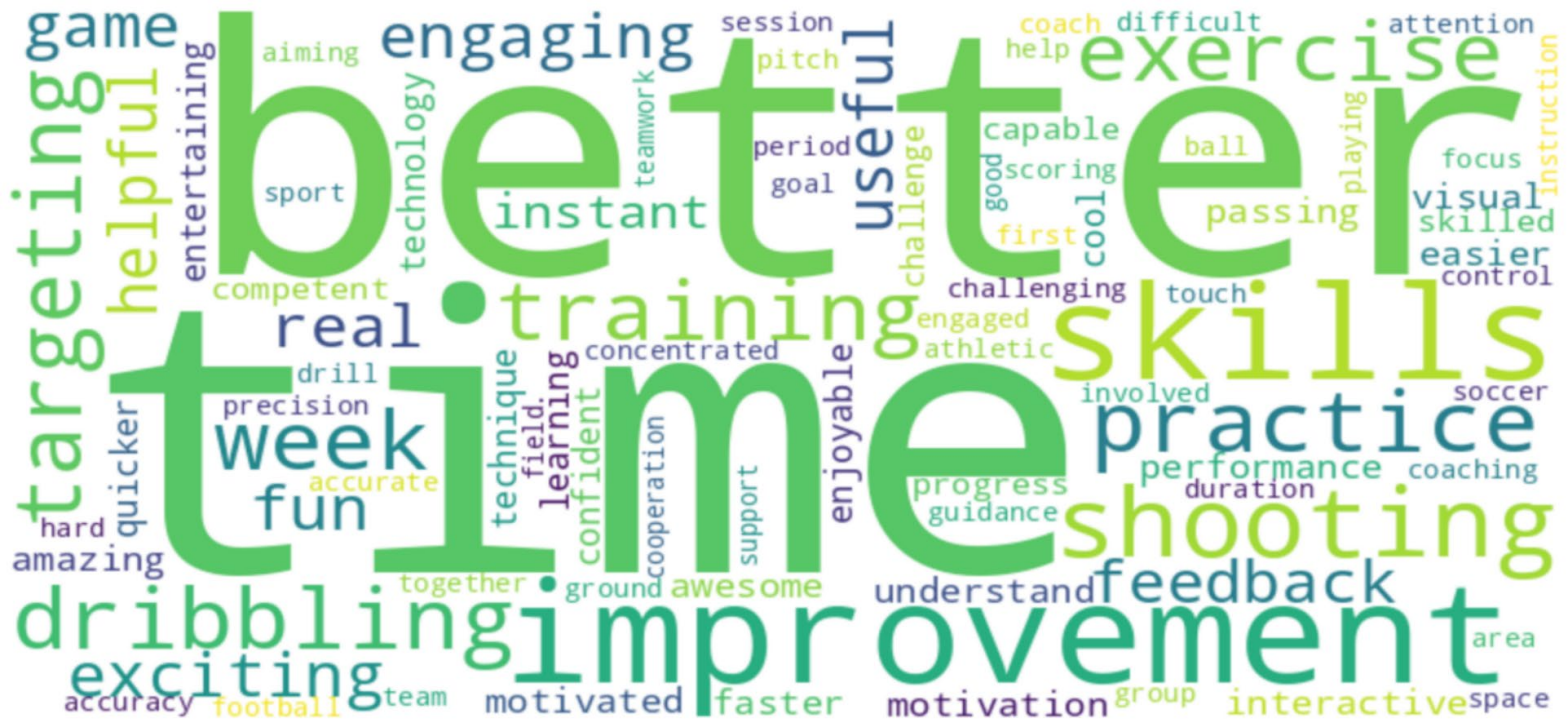
Word cloud of youth athlete comments on AR experience.

Thematic analysis of the semi-structured interviews revealed several key themes. Participants in the AR group reported that the feedback was “fun,” “helpful,” and “made them want to practice more.” They also commented on the “game-like” nature of the training, which they found to be highly engaging. Table 9 presents a thematic summary of the participant feedback, and Figure 11 shows a word cloud of the most frequently used words in the interviews. A word cloud was generated from the semi-structured interviews with participants in the AR group.

**Table 9:** Thematic summary of participant feedback.

Theme	Representative quotes
Engagement	“It was like playing a video game, but in real life.”
Usefulness	“The lines on the screen really helped me see where I was going wrong.”
Motivation	“I wanted to keep practicing to beat my high score.”
Challenge	“It was hard at first, but I got the hang of it.”

The results are correlated with the fact that AR feedback in real-time can considerably improve the performance of motor and intrinsic motivation in young athletes in comparison to traditional training. The AR group had superior marks in the dribbling, passing, and shooting skills, and also showed high marks in the retention tests. Also, the psychological involvement was improved among the athletes who obtained AR feedback on motivation subscales, interest, perceived competence, and effort. These trends were also confirmed by correlation analysis, which revealed that there were strong positive relationships between motivation and performance changes. All this implies that the AR-based training methodology can be applicable to enhance not just the acquisition of physical skills, but also motivation in sports with youth teams.

## 5 Discussion

This study was aimed at investigating the effects of real-time AR feedback on motor learning and intrinsic motivation in young athletes who play team sports. In particular, it was hypothesized in the study that AR feedback has greater effectiveness than conventional verbal coaching in enhancing motor performance, it has more effectiveness in increasing intrinsic motivation, and that any change in motivation is correlated to the increase in motor skill acquisition. Based on these purposes, we had the hypothesis that the AR feedback group would improve in motor performance and intrinsic motivation levels as compared to the control group, and that motivation and performance outcome would be positively correlated. The results are compared to the hypotheses proposed in the study and placed in the context of existing research in the area of motor learning, feedback, and sport motivation.

The results established that real-time AR feedback was more effective in enhancing motor performance based on all the measured skills than traditional coaching feedback. This upholds the hypothesis that AR-based feedback improves learning performance by offering real-time, accurate, and visually combined performance feedback. These findings can be correlated with other research showing that technology-based feedback is faster to correct errors and aid in the refinement of movement, especially in young athletes who acquire technical consistency.

The second hypothesis was also fulfilled as the AR feedback group had better scores in intrinsic motivation, especially in perceived competence and interest. Such inspirational benefits are aligned with the postulates of the SDT, which opine that competence supportive feedback promotes increased engagement and self-directed work effort. Although the research findings were measured in accordance with SDT constructs, mediating needs of autonomy, competence, and relatedness were not directly measured. The SDT-based instruments ought to be validated and used in future studies to understand these mechanisms. Examples are the Brain Electrophysiological recording and STimulation (BEST) toolbox, as suggested by Hassan et al. (2022) on systematic experimental design and quality control, standards of biomechanical and motion capture assessment indicated by Roupa et al. (2022), the protocols of data collection to wearable sensors introduced by Camomilla et al. (2018), and the systematic reporting framework of technological-based training intervention as suggested by O’Reilly et al. (2018) and Richter et al. (2024). Although our research was based on the use of AR feedback in real-world field settings, further research into the subject is welcome to design and report their study in such a way that it would be comparative, reproducible, and would integrate information from other studies. Adopting such standardized toolboxes would also strengthen the scientific credibility of AR-based sports training research and support the development of large-scale meta-analyses in this field. There was more engagement, interest, perceived competence, and effort during the intervention reported by the participants. The maintenance of high levels of these dimensions of motivation at follow-up indicates that, besides the short-term effects that AR feedback has on engagement, it may provide long-term motivational advantages. It particularly applies to youth sports, as fun and improvement are among the biggest motivators of children to keep playing sports. Interestingly, the observed associations with the increases in motivation and motor improvements are indicative of the fact that the two domains can be supportive of each other. Principles of cognitive and motor learning can be used to explain the effectiveness of AR feedback that was observed in the present study. The proximity between AR feedback allowed the learners to recognize and fix errors during the real performance, which reinforced the correlation between perception and action. The graphical data in the presented feedback gave the external representations that guided attention to the results of the tasks instead of focusing on the internal movement of the body, as the theory of attentional focus suggests. This is external attention, and thereby it decreased the cognitive load and allowed movement execution to be under automatic control.

Interestingly, AR feedback produced the most potent effects for discrete precision tasks such as shooting, while gains in continuous control tasks like dribbling were more modest. This differential pattern suggests that the immediacy and clarity of visual feedback may be particularly compelling for outcome-based actions, where movement correction can be applied in short, isolated attempts. In contrast, continuous skills require sustained attentional control, which may dilute the immediate influence of external visual cues. As most young athletes are under constant development and undergo continuous training, there is a possibility that constant participation in soccer is the reason why performance gains were maintained at the four-week mark. As such, the results can be attributed to early retention and not long-term consolidation. The future research must take into consideration the use of follow-up assessments over a period of several months to capture the long-term effects of augmented feedback on the long-term development of skills. The athletes who experienced the larger changes in motivational level also experienced the larger gains in skills, which may reflect the possible synergy between psychological involvement and the outcome of physical training. It is also necessary to note that part of the registered motivation growth could be related to novelty in the use of advanced technology. The interest and enthusiasm of working with AR tools may temporarily boost interest and self-reported pleasure.

This possible novelty effect is a natural reaction to technological innovation, yet it can be reduced with further exposure. Longitudinal studies or repeated training design should therefore be adopted in future research to determine whether the motivational and performance advantages of AR will be the same after the technology becomes well-known among the athletes. By tackling this factor, it will be possible to discern the long-term psychological and learning impacts as opposed to short-term induced responses of excitement. The current results can be discussed in terms of the propositions of the SDT because AR feedback could be used to increase motivation by providing better perceived competence, interest, and engagement with a task. It is, however, worth noting that the present design was not aimed at directly testing the mediating processes of SDT, i.e., the satisfaction of autonomy, competence, and relatedness needs that are key to the establishment of causal processes in the context of SDT. Thus, the motivational results here can be regarded as the adherence to SDT principles and not as conclusive ones.

### 5.1 Limitations and future research

Although the results of the research conducted in this paper offer valuable information on the importance of AR feedback in the sport training of youth, a number of limitations must be acknowledged. First, the intervention time was relatively short, and it was not possible to determine long-term retention and consolidation of motor learning. Longitudinal designs should also be used in future research to establish whether the recorded performance and motivation changes are maintained in the long term and at various phases of athletic growth. Second, the sample was a group of youth athletes that have been trained in one regional academy only, which could limit the application of the results to other sporting conditions or levels of competition. External validity would be enhanced by increasing the research in various institutions, different kinds of sports, and different ages. Third, the research was aimed at behavioral and self-report outcomes, and it was not concerned with direct measurement of underlying cognitive and neural processes. Future research may involve motion capture, eye-tracking, or neurophysiological research to learn more about how AR feedback affects the state of attentional focus, motor planning, and feedback processing. Lastly, although the motivational results agreed with the postulates of SDT, the current design failed to measure autonomy, competence, or relatedness as mediation variables. These mechanisms should be empirically tested in future research by using validated SDT-based need satisfaction scales. Overcoming these obstacles, future research will help to learn more about the role of AR technology in promoting motor learning and motivation, which will eventually allow for introducing more effective and evidence-based changes in the context of the implementation of digital tools into the youth sport training setting.

## 6 Conclusion

This paper has looked at how AR feedback that occurs in real-time affects motor and intrinsic motivation among youth athletes involved in team sports. These results have shown that AR feedback significantly improved motor performance, especially in discrete motor skills such as shooting and passing within a competitive feedback system, compared to the traditional feedback method. Besides that, athletes provided with AR feedback showed greater levels of intrinsic motivation, as well as significant changes in their perceived competence and enjoyment. These findings provide the indication that AR-mediated feedback has the potential to both foster technical skill growth and positively motivate individuals and, thus, allow promoting more effective learning experiences in the youth sport setting.

In practical terms, inclusion of AR feedback in the coach practice provides a prospective method to increase the engagement and learning effectiveness of young athletes. The urgency, accuracy, and visual responsiveness of AR technology allow the athletes to interpret feedback more efficiently and make amends with their actions in real-time, minimizing the reliance on the constant speech of the coach. As a coach and practitioner, the tools can be used as applicable supplements to the conventional techniques and can offer personalized and objective feedback that can be used to enhance the development of an athlete in a formal training setting. All these data contribute to the importance of AR technologies as an available, evidence-based intervention to promote motor learning and motivation in youth sport training.

## Data Availability

The original contributions presented in the study are included in the article/supplementary material, further inquiries can be directed to the corresponding author.

## References

[ref1] Al-Sinani Y. Al Taher M. (2023). Enhancing teaching skills of physical education teachers in the Sultanate of Oman through augmented reality strategies: a comprehensive feedback-based analysis. Cogent Soc. Sci. 9:2266253. doi: 10.1080/23311886.2023.2266253

[ref2] Balaguer I. Castillo I. Quested E. Duda J. L. (2013). “How do values relate to motivation?: values and motivational processes in youth sport: an AGT and SDT perspective” in Values in youth sport and physical education (London: Routledge, Taylor Francis), 119–133. doi: 10.4324/9780203114155

[ref3] Buchner J. Buntins K. Kerres M. (2022). The impact of augmented reality on cognitive load and performance: a systematic review. J. Comput. Assist. Learn. 38, 285–303. doi: 10.1111/jcal.12617

[ref4] Buszard T. (2022). On learning to anticipate in youth sport. Sports Med. 52, 2303–2314. doi: 10.1007/s40279-022-01694-z, PMID: 35622228 PMC9474538

[ref5] Calvo T. G. Cervelló E. Jiménez R. Iglesias D. Murcia J. A. M. (2010). Using self-determination theory to explain sport persistence and dropout in adolescent athletes. Span. J. Psychol. 13, 677–684. doi: 10.1017/S1138741600002341, PMID: 20977017

[ref6] Camomilla V. Bergamini E. Fantozzi S. Vannozzi G. (2018). Trends supporting the in-field use of wearable inertial sensors for sport performance evaluation: a systematic review. Sensors 18:873. doi: 10.3390/s18030873, PMID: 29543747 PMC5877384

[ref7] Cossich V. R. Carlgren D. Holash R. J. Katz L. (2023). Technological breakthroughs in sport: current practice and future potential of artificial intelligence, virtual reality, augmented reality, and modern data visualization in performance analysis. Appl. Sci. 13:12965. doi: 10.3390/app132312965

[ref8] Couture R. T. Singh M. Lee W. Chahal P. Wankel L. Oseen M. . (1999). Can mental training help to improve shooting accuracy? Policing. 22, 696–711. doi: 10.1108/13639519910299607

[ref9] Davaris M. Wijewickrema S. Zhou Y. Piromchai P. Bailey J. Kennedy G. . (2019). “The importance of automated real-time performance feedback in virtual reality temporal bone surgery training” in Artificial intelligence in education: 20th international conference, AIED 2019, Chicago, IL, USA, June 25–29, 2019, proceedings, part I. Switzerland AG: Springer, Cham. 20 96–109. doi: 10.1007/978-3-030-23204-7_9

[ref10] Dellaserra C. L. Gao Y. Ransdell L. (2014). Use of integrated technology in team sports: a review of opportunities, challenges, and future directions for athletes. J. Strength Cond. Res. 28, 556–573. doi: 10.1519/JSC.0b013e3182a952fb, PMID: 24263650

[ref11] Diamond A. (2012). Activities and programs that improve children’s executive functions. Curr. Dir. Psychol. Sci. 21, 335–341. doi: 10.1177/0963721412453722, PMID: 25328287 PMC4200392

[ref12] Diller F. Henkel N. Scheuermann G. Wiebel A. (2025). SkillAR: omnipresent in-situ feedback for motor skill training using AR. Virtual Reality 29, 1–11. doi: 10.1007/s10055-025-01108-1, PMID: 41272193

[ref13] Farley J. B. Stein J. Keogh J. W. Woods C. T. Milne N. (2020). The relationship between physical fitness qualities and sport-specific technical skills in female, team-based ball players: a systematic review. Sports Med Open 6, 1–20. doi: 10.1186/s40798-020-00245-y 32297147 PMC7158966

[ref14] Faruk M. Ali M. Susilana R. Dewi L. Alias N. Mahardika I. M. S. U. . (2025). The interventions of physical education by using augmented reality-based mobile learning can significantly improve gross motor skills in elementary school students. Retos 64, 201–210. doi: 10.47197/retos.v64.111727

[ref15] Fenton S. A. Duda J. L. Barrett T. (2016). Optimising physical activity engagement during youth sport: a self-determination theory approach. J. Sports Sci. 34, 1874–1884. doi: 10.1080/02640414.2016.1142104, PMID: 26873162

[ref16] Gallie D. Zhou Y. (2020). “Employee involvement, work engagement and skill development” in European Foundation for the Improvement of living and working conditions. Surrey Business School, University of Surrey, Guildford, England.

[ref17] Hassan U. Pillen S. Zrenner C. Bergmann T. O. (2022). The brain electrophysiological recording & stimulation (BEST) toolbox. Brain Stimul. 15, 109–115. doi: 10.1016/j.brs.2021.11.017, PMID: 34826626

[ref18] Hendry D. T. (2012). The role of developmental activities on self-determined motivation, passion, and skill in youth soccer players. Vancouver, BC: University of British Columbia.

[ref19] Huang Y.-C. Backman S. J. Backman K. F. McGuire F. A. Moore D. (2019). An investigation of motivation and experience in virtual learning environments: a self-determination theory. Educ. Inf. Technol. 24, 591–611. doi: 10.1007/s10639-018-9784-5

[ref20] Keegan R. J. Harwood C. G. Spray C. M. Lavallee D. E. (2009). A qualitative investigation exploring the motivational climate in early career sports participants: coach, parent and peer influences on sport motivation. Psychol. Sport Exerc. 10, 361–372. doi: 10.1016/j.psychsport.2008.12.003

[ref21] Kim J. W. Ritter F. E. Koubek R. J. (2013). An integrated theory for improved skill acquisition and retention in the three stages of learning. Theor. Issues Ergon. Sci. 14, 22–37. doi: 10.1080/1464536X.2011.573008

[ref22] Li X. Fan D. Feng J. Lei Y. Cheng C. Li X. (2025). Systematic review of motion capture in virtual reality: enhancing the precision of sports training. J. Ambient Intell. Smart Environ. 17, 5–27. doi: 10.3233/AIS-230198

[ref23] Lloyd R. S. Oliver J. L. Faigenbaum A. D. Howard R. Croix M. B. D. Williams C. A. . (2015). Long-term athletic development-part 1: a pathway for all youth. J. Strength Cond. Res. 29, 1439–1450. doi: 10.1519/JSC.0000000000000756 25486295

[ref24] Ma S. (2024). Revolutionizing sports education: harnessing innovations and technology for enhanced learning. Lect. Educ. Psychol. Public Media 53, 7–12. doi: 10.54254/2753-7048/53/20240012

[ref25] McKay A. K. Stellingwerff T. Smith E. S. Martin D. T. Mujika I. Goosey-Tolfrey V. L. . (2021). Defining training and performance caliber: a participant classification framework. Int. J. Sports Physiol. Perform. 17, 317–331. doi: 10.1123/ijspp.2021-0451 34965513

[ref26] Miles H. C. Pop S. R. Watt S. J. Lawrence G. P. John N. W. (2012). A review of virtual environments for training in ball sports. Comput. Graph. 36, 714–726. doi: 10.1016/j.cag.2012.04.007

[ref27] Ntoumanis N. Mallett C. J. (2014). “Motivation in sport: a self-determination theory perspective” in Routledge companion to sport and exercise psychology. Athanasios P. Dieter H. (Routledge, Taylor Francis), 67–82.

[ref28] O’Reilly M. Caulfield B. Ward T. Johnston W. Doherty C. (2018). Wearable inertial sensor systems for lower limb exercise detection and evaluation: a systematic review. Sports Med. 48, 1221–1246. doi: 10.1007/s40279-018-0878-4, PMID: 29476427

[ref29] Richter C. O’Reilly M. Delahunt E. (2024). Machine learning in sports science: challenges and opportunities. Sports Biomech. 23, 961–967. doi: 10.1080/14763141.2021.1910334 33874846

[ref30] Roupa I. da Silva M. R. Marques F. Gonçalves S. B. Flores P. da Silva M. T. (2022). On the modeling of biomechanical systems for human movement analysis: a narrative review. Arch. Comput. Methods Eng. 29, 4915–4958. doi: 10.1007/s11831-022-09757-0

[ref31] Ryan R. M. Deci E. L. (2000). Self-determination theory and the facilitation of intrinsic motivation, social development, and well-being. Am. Psychol. 55, 68–78. doi: 10.1037/0003-066X.55.1.68, PMID: 11392867

[ref32] Ryan R. M. Soenens B. Vansteenkiste M. (2019). Reflections on self-determination theory as an organizing framework for personality psychology: interfaces, integrations, issues, and unfinished business. J. Pers. 87, 115–145. doi: 10.1111/jopy.12440, PMID: 30325499

[ref33] Sattin D. Parma C. Lunetta C. Zulueta A. Lanzone J. Giani L. . (2023). An overview of the body schema and body image: theoretical models, methodological settings and pitfalls for rehabilitation of persons with neurological disorders. Brain Sci. 13:1410. doi: 10.3390/brainsci13101410, PMID: 37891779 PMC10605253

[ref34] Soltani P. Morice A. H. (2020). Augmented reality tools for sports education and training. Comput. Educ. 155:103923. doi: 10.1016/j.compedu.2020.103923

[ref35] Stone N. M. Kilding A. E. (2009). Aerobic conditioning for team sport athletes. Sports Med. 39, 615–642. doi: 10.2165/00007256-200939080-00002, PMID: 19769413

[ref36] Van Dam A. Eling P. Keijsers G. Becker E. (2013). Do employees with burnout prefer low-effort performance strategies? IIE Trans. Occup. Ergon. Hum. Factors 1, 190–201. doi: 10.1080/21577323.2013.828666

[ref37] Vansteenkiste M. Ryan R. M. Deci E. L., "Self-determination theory and the explanatory role of psychological needs in human well-being," Leuven, Belgium: University of Leuven, Belgium. 2006.

[ref38] Wilson A. D. Weightman A. Bingham G. P. Zhu Q. (2016). Using task dynamics to quantify the affordances of throwing for long distance and accuracy. J. Exp. Psychol. Hum. Percept. Perform. 42, 965–981. doi: 10.1037/xhp0000199, PMID: 26766510

[ref39] Wrisberg C. A. (2007). Sport skill instruction for coaches. Knoxville, Tennessee, United States: Human Kinetics.

[ref40] Wulf G. Shea C. Lewthwaite R. (2010). Motor skill learning and performance: a review of influential factors. Med. Educ. 44, 75–84. doi: 10.1111/j.1365-2923.2009.03421.x, PMID: 20078758

